# Association between postoperative thromboembolic and hemorrhagic complications and clinical outcomes after surgery for chronic subdural hematoma in patients with anticoagulation therapy for atrial fibrillation

**DOI:** 10.1007/s00701-024-06417-z

**Published:** 2025-01-16

**Authors:** Pihla Tommiska, Oula Knuutinen, Kimmo Lönnrot, Riku Kivisaari, Rahul Raj, on behalf of the FINISH study group, Abdirisak Ahmed, Tarmo Areda, Jiri Bartek, Tomasz Czuba, Nils Danner, Antti-Pekka Elomaa, Janek Frantzén, Ilkka Haapala, Joonas Haapasalo, Juuso Heikkilä, Minttu Hellman, Henna Henttonen, Nora Huuska, Teppo LN Järvinen, Henna-Kaisa Jyrkkänen, Aku Kaipainen, Olli-Pekka Kämäräinen, Hanna Kämppi, Milla Kelahaara, Riku Kivisaari, Nikolai Klimko, Oula A Knuutinen, Timo Koivisto, Tommi Korhonen, Janne Koskimäki, Anselmi Kovalainen, Xenia Kuparinen, Dan Laukka, Martin Lehecka, Kai Lehtimäki, Ville Leinonen, Kimmo Lönnrot, Antti Luikku, Teemu Luostarinen, Teemu Luoto, Janne Luotonen, Lauriina Lustig-Tammi, Henna-Riikka Maanpää, Jenni Määttä, Timo Möttönen, Eliisa Netti, Laura Nevaharju-Sarantis, Mika Niemelä, Tero Niskakangas, Mette Nissinen, Ville Nurminen, Minna Oinas, Teemu Ollonen, Anna Östberg, Elias Oulasvirta, Krista Pantzar, Katri Piilonen, Anni Pohjola, Markus Polvivaara, Jussi P Posti, Rahul Raj, Linnea Rajala, Jonas Ranstam, Minna Rauhala, Behnam Rezai Jahromi, Miika Roiha, Ilkka Saarenpää, Antti Sajanti, Henrikki Salmi, Jarno Satopää, Christoph Schwartz, Niina Shemeikka, Pia Sorto, Simo Taimela, Sami Tetri, Tuomo Thesleff, Pihla Tommiska, Maarit Tuomisto, Nuutti Vartiainen, Ville Vasankari, Jyri Virta, Mikko Visuri, Paula Walle, Frederick A Zeiler

**Affiliations:** 1 Department of Neurosurgery, Helsinki University Hospital and University of Helsinki, Haartmaninkatu 4, Po Box 320, 00029 HUS, Helsinki, Finland; 2 Department of Neurosurgery Neurocenter, Oulu University Hospital, Oulu, Finland; 3 Research Unit of Clinical Medicine, Faculty of Medicine, Oulu University Hospital and University of Oulu, Oulu, Finland; 4 Medical Research Center Oulu (MRC Oulu), University of Oulu, Oulu, Finland

**Keywords:** Chronic subdural hematoma, Atrial fibrillation, Anticoagulation medication, Thromboembolic complication, Hemorrhagic complication, Outcome

## Abstract

**Purpose:**

A substantial proportion of patients undergoing surgery for chronic subdural hematoma (CSDH) use anticoagulation medication due to atrial fibrillation (AF). We assessed the risk of postoperative thromboembolic and hemorrhagic complications in CSDH surgery patients with a history of anticoagulation for AF and their association with outcome.

**Methods:**

This posthoc analysis of a nationwide multicenter randomized controlled trial conducted during 2020–2022 included CSDH patients undergoing surgery with a history of preoperative anticoagulation use for AF. We assessed the incidence of thromboembolic and hemorrhagic complications and their associations with functional outcomes and mortality.

**Results:**

Of 589 patients, 128 patients (median age 83 years, 24% females) were on anticoagulation medication due to AF. The incidences of postoperative thromboembolic and hemorrhagic complications were 8% and 6%, respectively. A significantly higher proportion of patients with a thromboembolic complication had unfavorable functional outcome (70% vs. 21%, p < 0.001) and higher mortality (50% vs. 14%) than those without. After adjusting for risk factors, a thromboembolic complication was independently associated with a higher risk for unfavorable outcome (OR 16.8, 95% CI 3.0–94.2) and death (OR 11.1, 95% CI 2.4–52.0). Similarly, hemorrhagic complications associated independently with unfavorable outcome, although the effect size was smaller than for thromboembolic complications.

**Conclusion:**

The risk for thromboembolic complications seemed to be slightly higher than the risk for postoperative hemorrhagic complications after CSDH surgery in patients with a history of preoperative anticoagulation medication use due to AF. The occurrence of a thromboembolic complication was detrimental for patient prognosis, underscoring the importance of strategies to prevent thromboembolic events. There is an urgent need for a trial assessing the optimal timing of restarting anticoagulation medication after CSDH surgery.

**Trial registration:**

ClinicalTrials.gov identifier NCT04203550.

**Supplementary Information:**

The online version contains supplementary material available at 10.1007/s00701-024-06417-z.

## Introduction

The incidence of chronic subdural hematoma (CSDH) is increasing, projected to be the most common neurosurgical condition among adults in 2030, mainly due to an aging population and increased anticoagulation medication use [3, 10, 20, 33, 38, 48]. Up to 50–60% of CSDH patients use antithrombotic medications, primarily receiving anticoagulation therapy for atrial fibrillation (AF) [4, 20, 23, 24, 26, 30, 38, 47]. Standard practice for CSDH patients on anticoagulants involves pausing the medication preoperatively to prevent CSDH growth. However, no evidence-based guidelines exist for when to resume anticoagulation therapy postoperatively in patients with AF, making postoperative risk management challenging [7, 35, 45].

After elective surgery in patients with AF, when anticoagulation medication is resumed within 3 days, the risk of thromboembolic complications is as low as 0.3% [12]. Furthermore, when anticoagulation medication is resumed early (within 7 days) after ischemic stroke in patients with AF, the risk of thromboembolic complications is 1.8% [17]. In contrast, the reported incidence of postoperative thromboembolic complications after CSDH surgery is notably higher, ranging from 1.7% to 6.8% [1, 7, 18, 21, 26, 35].

Recent randomized controlled trials (RCTs) show varying protocols for resuming anticoagulation medication, ranging from 2 weeks [31] to 10 weeks [41]. The foremost reason for withholding postoperative anticoagulation medication is the fear of CSDH recurrence. However, whether continuation of anticoagulation medication really increases CSDH recurrence risk is controversial [1, 8, 18, 26, 33] and whether recurrence affects patient outcome is unclear [29, 37]. Although the risk of thromboembolic and hemorrhagic complications after CSDH surgery has been delineated before, their association with patient outcome has not been firmly established. Further, although middle meningeal artery (MMA) embolization is gaining popularity, where anticoagulation medication discontinuation might not be necessary, it is expected that surgery will remain essential for the urgent treatment of patients with CSDH who present with neurological symptoms [11, 16, 28].

This posthoc analysis of a national RCT assessed the risk of postoperative thromboembolic and hemorrhagic complications in CSDH patients with preoperative anticoagulation due to AF. We explored the association between these complications, functional outcome and death.

## Methods

### Study setting and population

We conducted a posthoc analysis of the FINISH trial including patients with a history of anticoagulation medication use due to AF [36]. Briefly, FINISH trial was an investigator-initiated, nationwide, randomized controlled, parallel-group, multicenter non-inferiority trial conducted in all five neurosurgical units in Finland from January 2020 to August 2022 and was registered on ClinicalTrials.gov (NCT04203550). In the trial, 589 patients were randomized to compare burr-hole drainage surgery with or without subdural irrigation on CSDH recurrences within 6 months of the index surgery. The trial showed that the risk for CSDH recurrence requiring reoperation was lower after the use of subdural irrigation with no differences in functional outcome or mortality between the groups. The study was approved by the ethics committee of Helsinki University Hospital. A written informed consent was obtained from all patients or their next-of-kin if the patient was unable to provide consent.

In the FINISH trial, anticoagulation medication was resumed after the routine 6 week control head computerized tomography (CT) scan [49]. The anticoagulation medication could be resumed earlier with reasonable clinical indication. The date of anticoagulation medication resumption was documented.

We obtained the data on all complications through phone interviews, questionnaires upon clinical follow-up visits, local picture archiving and communication systems (PACS), and through the nationwide electronic health record archive [25]. All complications were manually verified by the research team.

### Definition of thromboembolic and hemorrhagic complications

We defined a new postoperative thromboembolic complication as any arterial ischemic complication within 6 months of the index CSDH surgery. Arterial ischemic complications included ischemic stroke, myocardial infarction and systemic embolism (for example, mesenteric ischemia, lower limb ischemia) [39, 40, 46]. We did not define transient ischemic attacks as a thromboembolic complication [17].

We defined a new hemorrhagic complication as any new radiologically verified intracranial hemorrhage within 6 months of the index CSDH surgery. We considered the development of a new contralateral CSDH, or growth of an already existing contralateral CSDH as a hemorrhagic complication. We considered recurrence of the operated CSDH as a separate outcome and not as a hemorrhagic complication.

### Definition of functional outcome, mortality and the composite outcome

We assessed functional outcome using the modified Rankin Scale (mRS) score at 6 months after the index surgery (scale from 0 [no symptoms] to 6 [death]), with an mRS of 0–3 to represent favorable outcome and an mRS of 4–6 to represent unfavorable outcome. Trained research nurses and researchers, blinded to treatment allocation, assessed the mRS scores according to a standardized algorithm [6].

We assessed all-cause mortality within 6 months of the index surgery. We obtained the dates of death and causes of death through Statistics Finland. We defined a death as being vascular if the direct or contributing cause of death was vascular in nature (ICD-10 I*) [44].

We defined a composite outcome of thromboembolic complications, hemorrhagic complications or vascular deaths within 6 months of the index surgery.

### Definition of CSDH recurrence

The definition of CSDH recurrence was a recurrent symptomatic CSDH on the ipsilateral side requiring reoperation within 6 months of the index surgery [36].

### Statistical analysis

We reported medians with interquartile ranges (IQRs) for continuous data variables. For categorical variables, we reported numbers of observations and proportions. We compared categorical variables between groups using a two-sided chi-square test or a Fisher’s exact test (if the observed group value was ≤ 5). Continuous variables were compared using a Wilcoxon rank-sum test.

To assess the association between thromboembolic and hemorrhagic complications and outcome (mRS and mortality), we used logistic regression analysis, adjusting for covariates identified in univariate analysis as statistically significantly different (defined as a p-value < 0.05) between patients with a favorable and unfavorable 6-month functional outcome, as well as clinically relevant variables, including preoperative Glasgow Coma Scale (GCS) score and admission mRS score [5].

All analyses were conducted using Stata 18 (StataCorp). The Reporting of the study was done according to the STROBE cohort reporting guidelines [51].

### Results

**Fig. 1:**
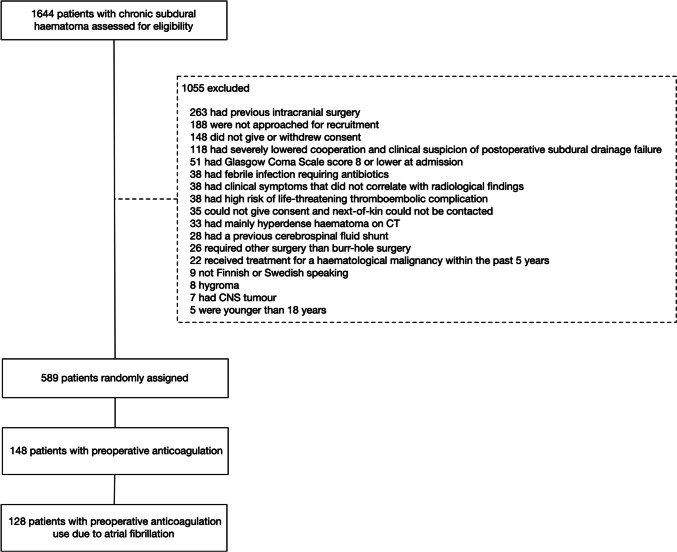
Flow chart. CT=computerized tomography, CNS=central nervous system

Of the 589 randomized patients, we included 128 patients who had a preoperative history of anticoagulation medication use due to AF (Fig. 1). Fifty-seven percent were using a direct oral anticoagulation, 41% warfarin and 3% low-molecular-weight heparin (one patient used both warfarin and low molecular weight heparin). Patients with anticoagulation medication use due to AF were older, had more comorbidities, lower admission GCS scores, higher admission mRS scores, and a higher degree of midline shift on the preoperative scan than all other patients without a history of anticoagulation medication due to AF (Table 1). There was no difference in CSDH recurrence rates between patients with a history of anticoagulation medication use due to AF and patients without a history of anticoagulation medication use due to AF (16% vs. 15%, p = 0.951, Table 2).

**Table 1:** Baseline characteristics for patients with a history of anticoagulation medication use due to atrial fibrillation versus patients without a history of anticoagulation medication use due to atrial fibrillation

Variable	Patients with anticoagulation medication due to AF (N = 128)	Patients without anticoagulation medication due to AF (N = 461)	p-value
Age, median (IQR), years	83 (78–89)	76 (70–83)	< 0.001
Female sex	31 (24%)	134 (29%)	0.280
Type of anticoagulant medication used prior to CSDH*			
DOAC	73 (57%)	0 (0%)	< 0.001
Warfarin	52 (41%)	15 (3%)	< 0.001
LMWH	4 (3%)	5 (1%)	0.096
Medical comorbidities†			
Diabetes mellitus	22 (17%)	106 (23%)	0.159
Previous cerebrovascular event	23 (18%)	61 (13%)	0.175
Hypertension	84 (66%)	256 (56%)	0.041
Ischemic heart disease or peripheral artery disease	24 (19%)	76 (16%)	0.546
Cardiac valve prosthesis	2 (2%)	0 (0%)	0.047
Pulmonary embolism or deep vein thrombosis‡	1 (1%)	12 (3%)	0.317
Dementia	25 (20%)	46 (10%)	0.003
History of head trauma			
Yes	100 (78%)	342 (74%)	0.568
No	12 (9%)	44 (10%)	
Unknown	16 (13%)	75 (16%)	
GCS at admission			
15	78 (61%)	387 (84%)	< 0.001
14	33 (26%)	55 (12%)	
9–13	17 (13%)	19 (4%)	
mRS score at admission			
1–3	63 (49%)	325 (70%)	< 0.001
4–5	65 (51%)	136 (30%)	
Hematoma laterality§			
Unilateral	99 (77%)	340 (74%)	0.430
Bilateral	29 (23%)	120 (26%)	
Midline shift, median (IQR), mm	7 (4–11)	6 (2–9)	0.011
Hematoma width¶, median (IQR), mm	23 (18–29)	24 (19–30)	0.183
Randomized group			
Irrigation	65 (51%)	229 (50%)	0.825
No irrigation	63 (49%)	232 (50%)	

^*^One patient had both warfarin and LMWH

^†^One patient can have several comorbidities

^‡^Medication used within 12 months before admission

^§^Patient can have a hematoma not operated on

^¶^Sum of left and right hematoma widths for bilateral hematomas

Abbreviations: *AF*, atrial fibrillation; *DOAC*, direct oral anticoagulant; *LMWH*, low molecular weight heparin; IQR, interquartile range; *GCS*, Glasgow Coma Scale; mRS, modified Rankin Scale; mm, millimeter

**Table 2:** Outcomes for patients with a history of anticoagulation medication use due to atrial fibrillation versus patients without a history of anticoagulation medication use due to atrial fibrillation

Variable	Patients with anticoagulation medication due to AF (N = 128)	Patients without anticoagulation medication due to AF (N = 461)	p-value
Thromboembolic complication	10 (8%)	6 (1%)	< 0.001
Hemorrhagic complication	8 (6%)	10 (2%)	0.018
CSDH recurrence	20 (16%)	71 (15%)	0.951
All-cause mortality	22 (17%)	17 (4%)	< 0.001
Vascular death	12 (9%)	6 (1%)	< 0.001
Composite outcome*	24 (19%)	20 (4%)	< 0.001

^*^Thromboembolic complications, hemorrhagic complications or vascular deaths within 6 months of the index surgery

#### Thromboembolic and hemorrhagic complications

The rate of thromboembolic complications (8% vs. 1%, *p* < 0.001) and hemorrhagic complications (6% vs. 2%, *p* = 0.018) were more frequent in patients with a preoperative history of anticoagulation medication use due to AF than in those without such a history (Table 2).

In the study population, the thromboembolic complications occurred after a median of 19 days (IQR 36–74) and the hemorrhagic complications occurred after a median of 31 days (IQR 1–86). Of the 8 hemorrhagic complications, 3 were procedure-related and occurred immediately after the index CSDH surgery (within 1 day of the operation), and 5 were delayed. The thromboembolic and hemorrhagic complications are listed in **eTable **1.

#### Resumption of anticoagulation medication

In 8 out of 10 patients (80%) with a thromboembolic complication, anticoagulation medication had not been restarted before the complication (**eFigure **1). In 2 out of 8 patients (25%) with a hemorrhagic complication, anticoagulation medication had been restarted before the complication.

#### All-cause mortality, vascular deaths and composite outcome

6-month all-cause mortality was higher in patients with a preoperative history of anticoagulation medication use due to AF compared to those without such a history (17% vs. 4%, *p* < 0.001, Table 2). Vascular death occurred in 9% of those with anticoagulation medication use due to AF compared to 1% in those without (*p* < 0.001). The composite outcome occurred in 19% of patients with a preoperative history of anticoagulation medication use due to AF compared to 4% in those without such a history (*p* < 0.001).

#### Functional outcome and mortality

**Fig. 2:**
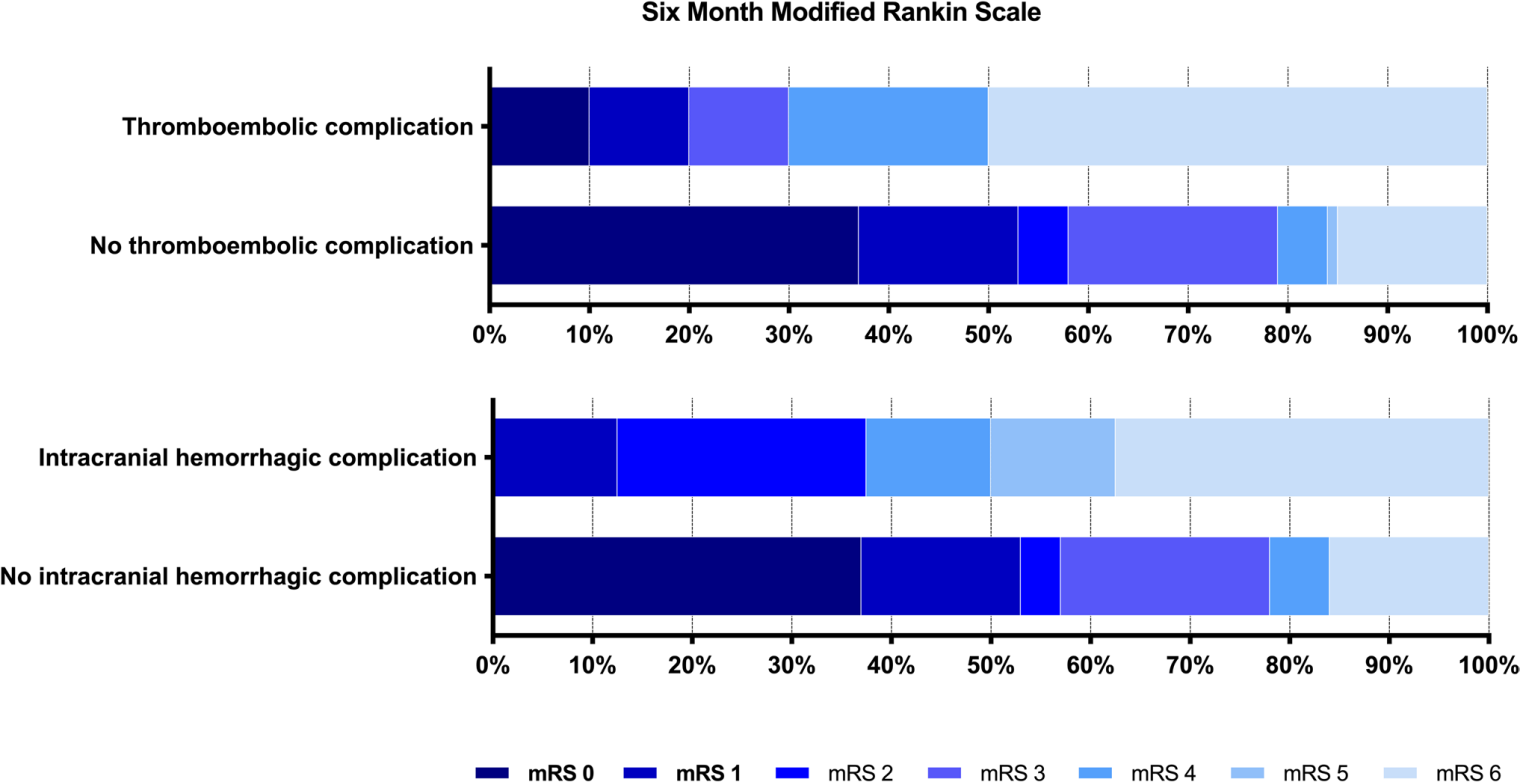
Modified Rankin Scale (mRS) score distribution 6 months after surgery for patients with and without thromboembolic and hemorrhagic complications. A score of 0 indicates no symptoms, 1 indicates no clinically significant disability despite symptoms, 2 indicates slight disability, 3 indicates moderate disability, 4 indicates moderately severe disability, 5 indicates severe disability, and 6 indicates death. mRS = modified Rankin Scale

The mRS was available for 125 out of 128 patients (98%) and mortality for all patients. A significantly higher proportion of patients with a thromboembolic complication had an unfavorable outcome (70% vs. 21%, p < 0.001) and higher mortality (50% vs. 14%, p = 0.004) than those without such a complication (Fig. 2, Table 3). A significantly higher proportion of patients with a hemorrhagic complication had poorer outcome than those without (Fig. 2, Table 3). CSDH recurrence was associated with a lower risk of unfavorable outcome.

**Table 3:** Outcome measures for patients with preoperative use of anticoagulation medication due to atrial fibrillation stratified by patients who had a thromboembolic or hemorrhagic complication versus those who did not have a thromboembolic or hemorrhagic complication

Outcome	mRS 0–3	mRS 4–6	p-value	Alive	Dead	p-value
Thromboembolic complication			0.001			0.004
No	91/115 (79%)	24/115 (21%)		101/118 (86%)	17/1118 (14%)	
Yes	3/10 (30%)	7/10 (70%)		5/10 (50%)	5/10 (50%)	
Hemorrhagic complication			0.011			0.116
No	91/117 (78%)	26/117 (22%)		101/120 (84%)	19/120 (16%)	
Yes	3/8 (38%)	5/8 (62%)		5/8 (63%)	3/8 (37%)	
CSDH recurrence			0.032			0.027
No	76/106 (72%)	30/106 (28%)		86/108 (80%)	22/108 (20%)	
Yes	18/19 (95%)	1/19 (5%)		20/20 (100%)	0/20 (0%)	
Composite outcome*			< 0.001			< 0.001
No	88/101 (87%)	13/101 (13%)		96/104 (92%)	8/104 (8%)	
Yes	6/24 (25%)	18/24 (75%)		10/24 (42%)	14/24 (58%)	

The modified Rankin Scale score was available for 125 out of 128 patients

^*^Thromboembolic complications, hemorrhagic complications or vascular deaths within 6 months of the index surgery

mRS, modified Rankin Scale

After adjusting for other risk factors in the multivariable logistic regression (age, history of dementia, midline shift on the preoperative scan, preoperative GCS score and preoperative mRS score, **eTable **2), the occurrence of a thromboembolic complication was associated with 16.8 times higher odds (95% CI 3.0–94.2, *p* = 0.001, **eTable **3) for unfavorable outcome and 11.1 times higher odds (95% CI 2.4–52.0, *p* = 0.002, **eTable **4) for death. The occurrence of a hemorrhagic complication was independently associated with a statistically significant increased odds for unfavorable outcome (OR 8.0, 95% CI 1.3–49.5, *p* = 0.025, **eTable **3) but not death (**eTable **4).

## Discussion

In this posthoc analysis of the FINISH trial, we showed that the incidence of thromboembolic complications was 8% and the incidence of hemorrhagic complications was 6% after CSDH surgery in adult patients with a history of anticoagulation use due to AF with a protocol of resuming anticoagulation medication 6 weeks after the surgery. Moreover, the composite outcome of thromboembolic complications, hemorrhagic complications or vascular death within 6 months was 19%. Patients suffering from a thromboembolic complication or hemorrhagic complication had a significantly higher risk of unfavorable outcome at 6 months. After adjusting for confounding factors, the occurrence of thromboembolic complications was independently associated with an unfavorable 6-month functional outcome and death. Similarly, the occurrence of a hemorrhagic complication was independently associated with unfavorable outcome, although the effect size was smaller than for thromboembolic complications. Patients with a preoperative history of anticoagulation medication due to AF did not have a higher risk for CSDH recurrence compared to those without such a history. Further, CSDH recurrence was associated with a lower risk of unfavorable outcome, most likely because patients who have died cannot undergo reoperation.

The main reason for withholding antithrombotic medication after CSDH surgery is to avoid a CSDH recurrence requiring reoperation. Several studies have looked at the association between preoperative use of antithrombotic medication and risk for CSDH recurrence, with conflicting results [1, 8, 9, 15, 18, 26, 32, 52]. However, it is important to realize that the goal of CSDH surgery is not only to prevent reoperations but also to improve neurological function. Thus, the entire therapeutic effect of continuing or withholding antithrombotic medication is not sufficiently captured by analyzing reoperation rates alone. Although preoperative anticoagulation medication appears to be a risk factor for the development of CSDH, the link between preoperative anticoagulation medication and CSDH recurrence is unclear. In line with several previous studies [9, 18, 26, 42, 50], we did not demonstrate an increased rate of CSDH recurrences in patients with preoperative anticoagulation due to AF compared to patients without preoperative anticoagulation due to AF (16% vs. 15%). Rather than being solely a hemorrhagic event, CSDH recurrence may represent a heightened local inflammatory response, potentially aggravated by the index surgery [14, 19, 43]. Thus, withholding anticoagulation medication to minimize the risk for hematoma recurrence might be futile and, possibly even harmful by increasing the risk for thromboembolic events.

In comparison to patients undergoing elective surgery, where the risk of thromboembolism in patients with AF is low (0.3–1.3%[12, 13, 27]), the incidence of thromboembolic events appears notably higher in patients undergoing surgery for CSDH [1, 7, 18, 21, 26, 35], being 8% in this study. Furthermore, the risk of thromboembolic events following CSDH surgery is higher compared to surgery for hip fractures, where the risk of ischemic stroke is estimated at 2.2–2.5% [22, 34], with previously diagnosed AF being a major risk factor [2, 22]. Additionally, the risk of the composite outcome of thromboembolic complication, hemorrhagic complication or vascular death seems to be notably higher in CSDH patients than in patients with ischemic stroke (19% vs. 2.9–4.1%[17]). A likely reason for the discrepancy in the frequency of thromboembolic and hemorrhagic events is the non-evidence-based, heterogeneous practices regarding the resumption of anticoagulation medication after CSDH surgery.

The question remains whether the additional risks of thromboembolic and hemorrhagic complications after CSDH surgery can be avoided by optimizing the timing of restarting the anticoagulation medication. Based on our findings, the risk for hemorrhagic complications might not be avoidable by changing postoperative anticoagulation medication strategy, as there were only 2 hemorrhagic complications that occurred shortly after restarting the medication. In contrast, anticoagulation medication was not restarted before the thromboembolic complication in most cases. Furthermore, since CSDH recurrence may not significantly impact patient outcomes, early resumption of anticoagulation medication after CSDH surgery could potentially prevent some thromboembolic events, leading to improved outcomes even if the risk of CSDH recurrence increases [29, 37]. Thus, similar to ischemic stroke, it seems plausible that a more aggressive approach to resuming anticoagulation medication after CSDH surgery may be a viable option [17].

### Limitations

This was a posthoc analysis of an RCT that was designed to answer a different research question. As such, there is potential for bias, particularly due to unmeasured confounders, including patient comorbidities that may influence the risk of thromboembolic and bleeding events, the type of anticoagulation medication used, and patient-specific factors such as smoking status. Thus, our results should be interpreted as hypothesis generating. Noteworthy is that this study did not try to, nor does it show when anticoagulants are safe to resume after CSDH surgery. The absolute number of thromboembolic and hemorrhagic complications was relatively small, even though the study population represents one of the largest RCTs conducted on CSDH patients [36]. The sample size was therefore insufficient to assess the association between early and late complications and patient outcomes. The designed study protocol stated that anticoagulation for patients with AF would only be started after 6 weeks of the index surgery following a routine postoperative CT. Thus, the generalizability of the results to settings with different protocols regarding antithrombotic medication continuation may be hampered. Further, we did not collect data regarding when the anticoagulation medication was preoperatively paused. It is possible that some of the thromboembolic complications are the result of preoperative discontinuation of the medication rather than the postoperative pause. Further, the emerging popularity of MMA embolization might reduce the clinical problem of anticoagulation medication discontinuation in CSDH patients. However, surgery will continue to play a crucial role in the emergent treatment of patients with CSDH presenting with neurological symptoms.

## Conclusion

After CSDH surgery, the risk for thromboembolic complications was 8%, and the risk for postoperative hemorrhagic complications was 6% in patients with a history of preoperative anticoagulation medication use due to AF. Postoperative thromboembolic complications were independently associated with poorer outcomes, underscoring the importance of strategies to prevent thromboembolic complications. Early restart of anticoagulation medication might decrease the risk for postoperative thromboembolic complications, while potentially not increasing the risk of hemorrhagic complications after CSDH surgery. There is an urgent need for a trial assessing the optimal timing of restarting anticoagulation medication after CSDH surgery.

## Supplementary Information

Below is the link to the electronic supplementary material.

ESM 1 (DOCX 119 KB )

## Data Availability

All data requests should be submitted to RR by e-mail for consideration. Access to anonymized data may be granted following review and consideration of the Act on the Secondary Use of Health and Social Data in Finland.
