# Supplementary material for: Association between postoperative thromboembolic and hemorrhagic complications and clinical outcomes after surgery for chronic subdural hematoma in patients with anticoagulation therapy for atrial fibrillation
Source: Acta Neurochir (Wien). 2025 Jan 16;167(1):17. doi: 10.1007/s00701-024-06417-z (PMC11735564; doi:10.1007/s00701-024-06417-z)
Supplement: Supplementary file 1 — (DOCX 119 KB ) [file 701_2024_6417_MOESM1_ESM.docx]

**Complete list of FINISH investigators**

| **First and middle names or initials** | **Surnames** |
| --- | --- |
| Abdirisak | Ahmed |
| Tarmo | Areda |
| Jiri Jr | Bartek |
| Tomasz | Czuba |
| Nils | Danner |
| Antti-Pekka | Elomaa |
| Janek | Frantzén |
| Ilkka | Haapala |
| Joonas | Haapasalo |
| Juuso | Heikkilä |
| Minttu | Hellman |
| Henna | Henttonen |
| Nora | Huuska |
| Teppo LN | Järvinen |
| Henna-Kaisa | Jyrkkänen |
| Aku | Kaipainen |
| Olli-Pekka | Kämäräinen |
| Hanna | Kämppi |
| Milla | Kelahaara |
| Riku | Kivisaari |
| Nikolai | Klimko |
| Oula A | Knuutinen |
| Timo | Koivisto |
| Tommi | Korhonen |
| Janne | Koskimäki |
| Anselmi | Kovalainen |
| Xenia | Kuparinen |
| Dan | Laukka |
| Martin | Lehecka |
| Kai | Lehtimäki |
| Ville | Leinonen |
| Kimmo | Lönnrot |
| Antti | Luikku |
| Teemu | Luostarinen |
| Teemu | Luoto |
| Janne | Luotonen |
| Lauriina | Lustig-Tammi |
| Henna-Riikka | Maanpää |
| Jenni | Määttä |
| Timo | Möttönen |
| Eliisa | Netti |
| Laura | Nevaharju-Sarantis |
| Mika | Niemelä |
| Tero | Niskakangas |
| Mette | Nissinen |
| Ville | Nurminen |
| Minna | Oinas |
| Teemu | Ollonen |
| Anna | Östberg |
| Elias | Oulasvirta |
| Krista | Pantzar |
| Katri | Piilonen |
| Anni | Pohjola |
| Markus | Polvivaara |
| Jussi P | Posti |
| Rahul | Raj |
| Linnea | Rajala |
| Jonas | Ranstam |
| Minna | Rauhala |
| Behnam | Rezai Jahromi |
| Miika | Roiha |
| Ilkka | Saarenpää |
| Antti | Sajanti |
| Henrikki | Salmi |
| Jarno | Satopää |
| Christoph | Schwartz |
| Niina | Shemeikka |
| Pia | Sorto |
| Simo | Taimela |
| Sami | Tetri |
| Tuomo | Thesleff |
| Pihla | Tommiska |
| Maarit | Tuomisto |
| Nuutti | Vartiainen |
| Ville | Vasankari |
| Jyri | Virta |
| Mikko | Visuri |
| Paula | Walle |
| Frederick A | Zeiler |

| **eTable 1**: Types and frequencies of thromboembolic and hemorrhagic complications after CSDH surgery in patients with a history of anticoagulation medication use due to atrial fibrillation | |
| --- | --- |
| **Thromboembolic complication** | **Frequency (N)** |
| Cerebral infarction | 7 |
| Mesenteric ischemia | 3 |
| **Hemorrhagic complication** | **Frequency (N)** |
| Acute subdural hematoma | 3 |
| Subarachnoid hemorrhage | 2 |
| Intracerebral hemorrhage | 2 |
| Significant growth of contralateral CSDH | 1 |
| Abbreviations: CSDH, chronic subdural hematoma. | |

| **eTable 2**: Univariate association risk factor differences between patients with a favorable functional outcome (modified Rankin Scale 0–3) and unfavorable functional outcome (modified Rankin Scale 4–6) at 6 months including patients only with preoperative use of anticoagulation medication due to atrial fibrillation. | | | |
| --- | --- | --- | --- |
| **Variable** | **Modified Rankin Scale 0–3 (N = 94)** | **Modified Rankin Scale 4–6 (N = 31)** | ***P* value** |
| **Age**, median (IQR), years | 82 (76–87) | 88 (82–90) | <0.001 |
| **Female sex** | 133 (27%) | 23 (32%) | 0.407 |
| **Medical comorbidities*** |  |  |  |
| Diabetes mellitus | 15 (16%) | 6 (19%) | 0.661 |
| Previous cerebrovascular event | 13 (13%) | 8 (26%) | 0.122 |
| Hypertension | 61 (65%) | 20 (65%) | 0.970 |
| Ischemic heart disease or peripheral artery disease | 16 (17%) | 8 (26%) | 0.282 |
| Cardiac valve prosthesis | 1 (1%) | 1 (3%) | 0.436 |
| Pulmonary embolism or deep vein thrombosis† | 1 (1%) | 0 (0%) | 0.752 |
| Dementia | 9 (10%) | 14 (45%) | <0.001 |
| **History of head trauma** |  |  |  |
| Yes | 72 (77%) | 26 (84%) | 0.681 |
| No | 10 (11%) | 2 (6%) |  |
| Unknown | 12 (12%) | 3 (10%) |  |
| **GCS at admission** |  |  |  |
| 15 | 63 (67%) | 15 (48%) | 0.097 |
| 14 | 22 (23%) | 9 (29%) |  |
| 9–13 | 9 (10%) | 7 (23%) |  |
| **mRS score at admission** |  |  |  |
| 1–3 | 49 (52%) | 14 (45%) | 0.501 |
| 4–5 | 45 (48%) | 17 (55%) |  |
| **Hematoma laterality‡** |  |  |  |
| Unilateral | 72 (77%) | 26 (84%) | 0.393 |
| Bilateral | 22 (23%) | 5 (16%) |  |
| **Midline shift**, median (IQR), mm | 8 (4–12) | 6 (4–7) | 0.021 |
| **Hematoma width**,§ median (IQR), mm | 24 (18–29) | 21 (16–27) | 0.128 |
| **Randomized group** |  |  |  |
| Irrigation | 46 (49%) | 17 (55%) | 0.569 |
| No irrigation | 48 (51%) | 14 (45%) |  |
| **Thromboembolic complication** |  |  | 0.001 |
| No | 91 (97%) | 24 (77%) |  |
| Yes | 3 (3%) | 7 (23%) |  |
| **Hemorrhagic complication** |  |  | 0.011 |
| No | 91 (97%) | 26 (84%) |  |
| Yes | 3 (3%) | 5 (16%) |  |
| **CSDH reoperation** |  |  |  |
| No | 76 (81%) | 30 (97%) | 0.032 |
| Yes | 18 (19%) | 1 (3%) |  |
| The modified Rankin Scale score was available for 125 out of 128 patients.  *One patient can have several comorbidities.  †Medication used within 12 months before admission.  ‡Patient can have a hematoma not operated on.  §Sum of left and right hematoma widths for bilateral hematomas.  Abbreviations: IQR, interquartile range; GCS, Glasgow Coma Scale; mRS, modified Rankin Scale; mm, millimeter. | | | |

| **eTable 3:** Multivariable logistic regression model showing the association between postoperative thromboembolic and hemorrhagic complications and other risk factors and the dichotomized modified Rankin Scale score 4–6 vs. 0–3 at 6 months. | | | |
| --- | --- | --- | --- |
| **Variable** | **Odds ratio** | **95% CI** | ***P* value** |
| **Age** | 1.07 | 0.98–1.17 | 0.120 |
| **Preoperative diagnosis of dementia** | 4.41 | 1.30–14.95 | 0.017 |
| **Midline shift (mm)** | 0.88 | 0.78–1.00 | 0.048 |
| **mRS score at admission** | 0.99 | 0.61–1.63 | 0.983 |
| **GCS at admission** | 0.63 | 0.35–1.12 | 0.116 |
| **Thromboembolic complication** |  |  |  |
| No | 1.0 |  |  |
| Yes | 16.75 | 2.98–94.21 | 0.001 |
| **Hemorrhagic complication** |  |  |  |
| No | 1.0 |  |  |
| Yes | 8.02 | 1.30–49.50 | 0.025 |
| The modified Rankin Scale score was available for 125 out of 128 patients.  Abbreviations: CI, Confidence Interval; GCS, Glasgow Coma Scale; mRS, modified Rankin Scale.  Study site was used as the random effect.  The model’s mean variance inflation factor was 1.25 and the maximal variance inflation factor was 1.54 indicating no or low collinearity. | | | |

| **eTable 4:** Multivariable logistic regression model showing the association between postoperative thromboembolic and hemorrhagic complications and other risk factors and dead vs. alive at 6 months. | | | |
| --- | --- | --- | --- |
| **Variable** | **Odds ratio** | **95% CI** | ***P* value** |
| **Age** | 1.08 | 0.98–1.18 | 0.108 |
| **Preoperative diagnosis of dementia** | 1.93 | 0.55–6.83 | 0.306 |
| **Midline shift (mm)** | 0.92 | 0.81–1.04 | 0.168 |
| **mRS score at admission** | 0.85 | 0.51–1.40 | 0.517 |
| **GCS at admission** | 0.63 | 0.37–1.07 | 0.089 |
| **Thromboembolic complication** |  |  |  |
| No | 1.0 |  |  |
| Yes | 7.08 | 1.53–32.67 | 0.012 |
| **Hemorrhagic complication** |  |  |  |
| No | 1.0 |  |  |
| Yes | 4.19 | 0.79–22.29 | 0.093 |
| The modified Rankin Scale score was available for 125 out of 128 patients.  Abbreviations: CI, Confidence Interval; GCS, Glasgow Coma Scale; mRS, modified Rankin Scale.  Study site was used as the random effect.  The model’s mean variance inflation factor was 1.26 and the maximal variance inflation factor was 1.55 indicating no or low collinearity. | | | |


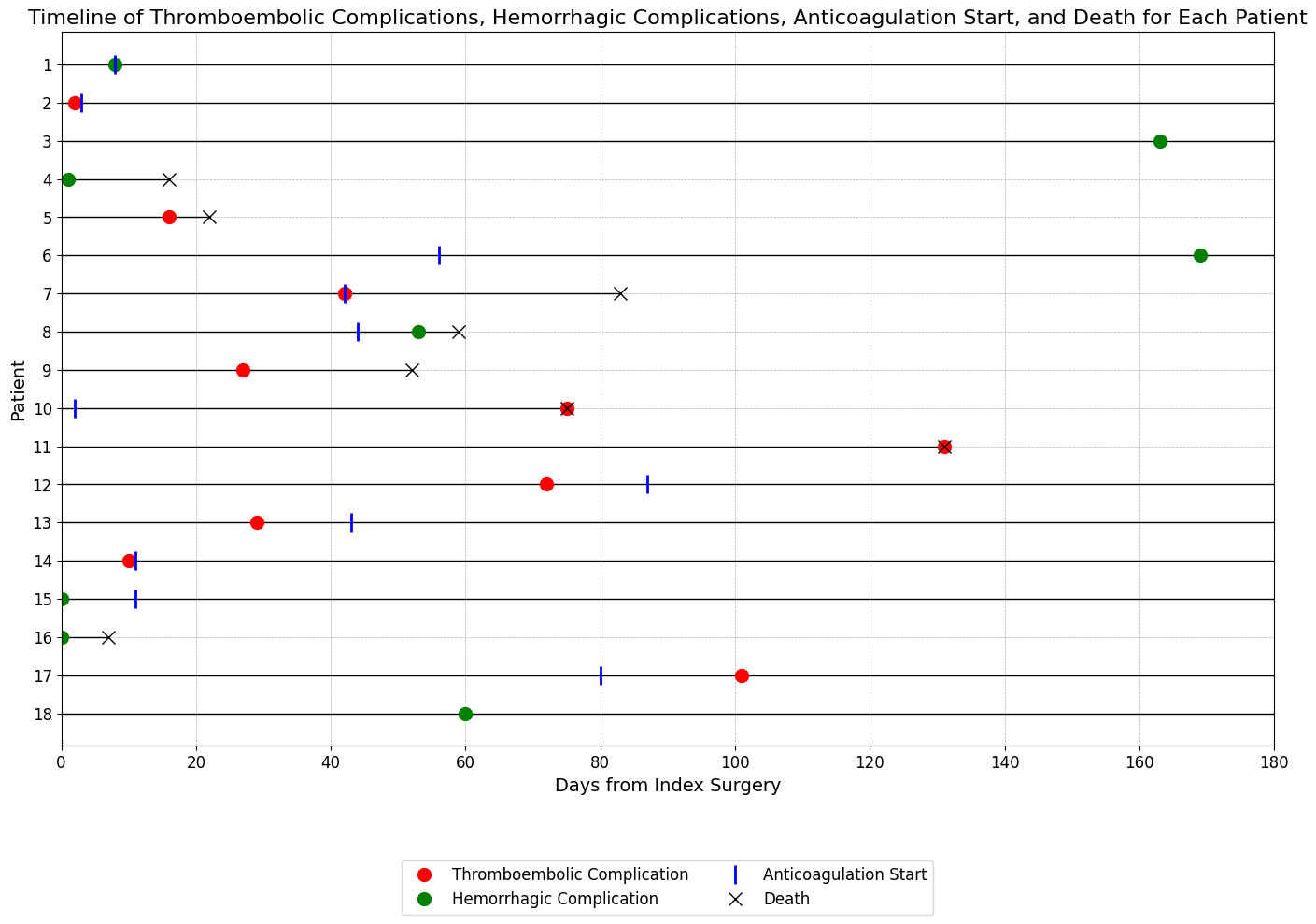


**eFigure 1**: The timeline following chronic subdural hematoma surgery for each patient with a postoperative thromboembolic complication and/or an intracranial hemorrhagic complication, and the relationship between restart of the anticoagulation medication and the complication. All these patients had a preoperative history of anticoagulation medication use due to atrial fibrillation.
